# Patient-Reported Outcome (PRO) Assessment in Clinical Trials: A Systematic Review of Guidance for Trial Protocol Writers

**DOI:** 10.1371/journal.pone.0110216

**Published:** 2014-10-15

**Authors:** Melanie Calvert, Derek Kyte, Helen Duffy, Adrian Gheorghe, Rebecca Mercieca-Bebber, Jonathan Ives, Heather Draper, Michael Brundage, Jane Blazeby, Madeleine King

**Affiliations:** 1 Primary Care and Clinical Sciences, University of Birmingham, Birmingham, United Kingdom; 2 MRC Midland Hub for Trials Methodology Research, Birmingham, United Kingdom; 3 School of Sport, Exercise & Rehabilitation, University of Birmingham, Birmingham, United Kingdom; 4 Department of Global Health and Development, London School of Hygiene & Tropical Medicine, London, United Kingdom; 5 Psycho-oncology Co-operative Research Group, University of Sydney, Sydney, Australia; 6 Medicine. Ethics, Society and History, University of Birmingham, Birmingham, United Kingdom; 7 Queens University, Kingston, Ontario, Canada; 8 MRC ConDuCT II Hub for Trials Methodology Research, School of Social & Community Medicine, University of Bristol, Bristol, United Kingdom; University Hospital Basel, Switzerland

## Abstract

**Background:**

Evidence suggests there are inconsistencies in patient-reported outcome (PRO) assessment and reporting in clinical trials, which may limit the use of these data to inform patient care. For trials with a PRO endpoint, routine inclusion of key PRO information in the protocol may help improve trial conduct and the reporting and appraisal of PRO results; however, it is currently unclear exactly what PRO-specific information should be included. The aim of this review was to summarize the current PRO-specific guidance for clinical trial protocol developers.

**Methods and Findings:**

We searched the MEDLINE, EMBASE, CINHAL and Cochrane Library databases (inception to February 2013) for PRO-specific guidance regarding trial protocol development. Further guidance documents were identified via Google, Google scholar, requests to members of the UK Clinical Research Collaboration registered clinical trials units and international experts. Two independent investigators undertook title/abstract screening, full text review and data extraction, with a third involved in the event of disagreement. 21,175 citations were screened and 54 met the inclusion criteria. Guidance documents were difficult to access: electronic database searches identified just 8 documents, with the remaining 46 sourced elsewhere (5 from citation tracking, 27 from hand searching, 7 from the grey literature review and 7 from experts). 162 unique PRO-specific protocol recommendations were extracted from included documents. A further 10 PRO recommendations were identified relating to supporting trial documentation. Only 5/162 (3%) recommendations appeared in ≥50% of guidance documents reviewed, indicating a lack of consistency.

**Conclusions:**

PRO-specific protocol guidelines were difficult to access, lacked consistency and may be challenging to implement in practice. There is a need to develop easily accessible consensus-driven PRO protocol guidance. Guidance should be aimed at ensuring key PRO information is routinely included in appropriate trial protocols, in order to facilitate rigorous collection/reporting of PRO data, to effectively inform patient care.

## Introduction

Patient-reported outcomes (PROs), including health-related quality of life (HRQL), symptoms such as pain or fatigue, and health utility, are increasingly assessed in clinical trials as a measure of effectiveness.[1]–[3] PRO trial data may be used to inform clinical care and decision-making, predict long-term outcomes and influence health-policy; but to do so, as with any trial outcome, they must be collected with rigor. Unfortunately, evidence shows that the quality of PRO data can be undermined in some trials by inconsistencies in data collection [4] and, in particular, by high rates of missing data [5]; this adversely affects the integrity and usefulness of such data in clinical practice.

To help ensure optimal PRO data collection, PRO-specific components should be considered during clinical trial design and clearly documented in the trial protocol. [6], [7] The trial protocol is the cornerstone of a well-conducted trial, and should provide specific instruction on how to conduct all aspects of the study. [8] The protocol also allows external funding bodies, regulators, research ethics committees, journal editors, health care providers, systematic reviewers and policy makers to evaluate the design and methods. [6] Despite the importance of PROs, recent data suggests that some trial staff feel protocols provide little guidance regarding PRO-specific aspects of the trial, leading to ambiguity and the potential for significant inconsistency in the way PRO data are gathered, analysed, acted upon, and reported. [4], [9], [10].

The recent publication of the SPIRIT (Standard Protocol Items: Recommendations for Interventional Trials) guidance aims to promote the inclusion of important *general* methodological components in trial protocols [6]; however, it does not provide specific guidance related to PROs. It is currently unclear exactly what PRO-specific information should be included in trial protocols. The aim of this systematic review was to summarize current PRO-specific guidance for clinical trial protocol developers.

## Methods

### Ethics

This study received ethical approval from the University of Birmingham ethical review board (ERN_13-0047).

This review was conducted according to PRISMA guidelines (Checklist S1) [11] and a protocol is available. [12] Given the methodological focus of the review this was not registered with PROSPERO at http://www.crd.york.ac.uk/PROSPERO/.

### Eligibility criteria

Papers were deemed eligible if they contained guidance (in the form of advice or formal recommendations) and/or a checklist on PRO related trial protocol content. As PROs is an ‘umbrella term’ papers including protocol guidance relating to specific types of PRO (such as HRQL) were also deemed eligible.

### Information Sources and Search Strategy

The MEDLINE (Ovid), EMBASE, CINHAL and Cochrane Library databases were searched from inception to February 2013 (electronic search strategies are presented in full in Appendix S1). Other relevant articles were identified via two Internet search engines (Google and Google Scholar) using the key words ‘Patient-Reported Outcomes’ or ‘Health-Related Quality of Life’ in combination with ‘Guidance’, ‘Guidelines’ or ‘Checklist’. Only the first 30 results (3 pages) of each search were reviewed as article relevance diminishes substantially with each page of results. [13] In addition, an international advisory group (MB, AG, JB, RMB and MK) were consulted via email to identify additional ‘grey literature’ directly relevant to the research question. Finally, PRO guidance/checklists and Standard Operating Procedures (SOPs) were requested from all members of the UK Clinical Research Collaboration registered clinical trials units (UK CRC-CTU) via email, with one follow up reminder. All citations were downloaded into Endnote software version X7 and duplicates deleted. Records were then screened by title/abstract before full-text articles/documents were retrieved for eligibility evaluation. Remaining articles were subject to a citation search before a final hand-search of all reference lists.

### Guidance selection

Papers and other guidance documents were deemed eligible if they provided guidance (advice or formal recommendations) and/or a checklist describing key PRO-specific information that should be specified in clinical trial protocols. Non-English papers were screened by language specialists in the School of Health and Population Sciences, University of Birmingham. When more than one edition of a book was available, the latest edition was screened.

### Data Extraction

Two reviewers (HDu and AG) independently screened the titles and abstracts of all citations. Full text versions of potentially eligible documents were independently reviewed (HDu and AG) with uncertainties resolved through discussion with a third investigator (MC/DK). Two investigators (MC and DK), independently and in duplicate, extracted both the publication details and all PRO-specific protocol recommendations from the final included documents. Data were extracted into Excel on pre-specified forms to capture: publication source, year of publication, clinical and regulatory focus. Each recommendation was identified through independent review by MC and DK and iteratively added to the spreadsheet following checks for consistency.

Both explicit (‘stated clearly and in detail, leaving no room for confusion or doubt’ [14]) and implicit (‘suggested though not directly expressed’ [14]) recommendations for PRO related protocol content were extracted. Explicit recommendations specifically stated that an item of PRO information should be included in the trial protocol. Implicit recommendations described important PRO trial design issues, and were written in such a way as to suggest items should be included in the protocol – without specifically stating so.

### Summary of Guidance

For ease of interpretation, PRO protocol recommendations were extracted and grouped as per SPIRIT guidance headings. [6] Duplicate recommendations within each of these sections were identified by MC and DK, and were merged where necessary following discussion with the international advisory group. The proportion of guidance documents associated with each recommendation was identified. To assess general trends in guidance over time, the proportion of guidance documents per recommendation was analysed retrospectively over 5 year time periods.

## Results

### Selection of Guidance Documents

**Figure 1:**
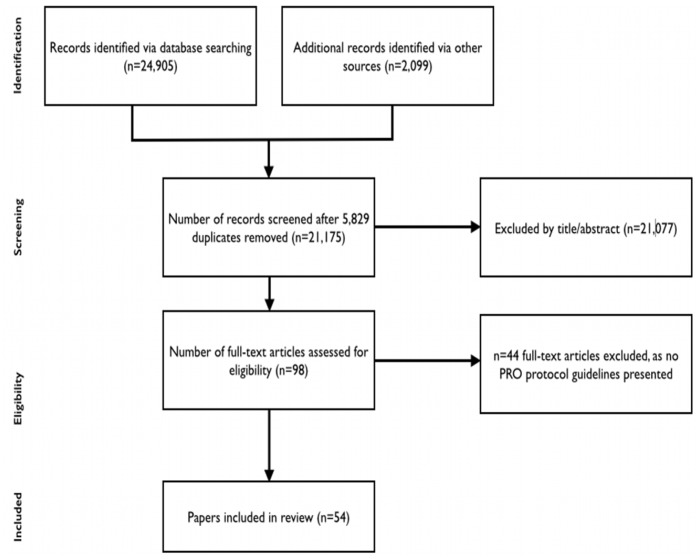
Search results flow diagram.

The literature search yielded 21,175 unique references. Following application of the inclusion criteria, 54 guidance documents[1], [15]–[67] were included in the review (Figure 1). DK and MC independently included 53 of the 54 articles. One paper was initially excluded by MC but later included following discussion by the research team. [52] Of the 54 included documents, 8 were identified from searches of electronic databases, 5 from citation tracking, 27 from hand searching the reference lists of included articles, 7 from the grey literature review and 7 from expert recommendations.

### Guidance Characteristics

Document characteristics are summarised in Table 1. The included materials dated from 1989 to 2013 and included 42 journal articles, 5 books and 7 organizational guideline documents, with the majority focused on HRQL/PRO assessment in cancer trials (n = 35, 64.8%) and written from a non-regulatory perspective (n = 44, 81.5%).

**Table 1:** Guidance Document Characteristics.

Authors	Year	Clinical area inwhich guidanceis focused.	PRO Protocol Checklist provided	RegulatoryFocus*	Source
Moinpour et al. [15]	1989	Oncology			Journal of the National Cancer Institute
Schron & Schumaker [16]	1992	Cardiovascular			Progress in cardiovascular Nursing
Gotay et al. [17]	1992a	Oncology			Journal of the National cancer Institute
Osoba [18]	1992	Oncology	Yes		Quality of Life Research
Gotay et al. [19]	1992b	Oncology			Oncology
Nayfield et al. [20]	1992	Oncology			Quality of Life Research
Sadura et al. [21]	1992	Oncology			Journal of the National Cancer Institute
Hayden et al. [22]	1993	Oncology			Oncology Nursing Forum
Cella et al. [23]	1993	General			Quality of Life research
Spilker [24]	1996	General			Book (Chapters 45 and 72)
Molin & Arrigo [25]	1995	Oncology	Yes		European Journal of Cancer
Fayers et al. [26]	1997	Oncology	Yes		European Journal Cancer
Kiebert [27]	1997	Oncology	Yes		European Journal of Cancer
Bernhard et al. [28]	1998a	Oncology			Statistics in medicine
Bernhard et al. [29]	1998b	Oncology			Statistics in medicine
Osoba [30]	1998	Oncology			Statistics in Medicine
Simes et al. [31]	1998	Oncology			Statistics in medicine
Moinpour & Lovato [32]	1998	Oncology			Statistics in Medicine
Brooks et al. [33]	1998	Cardiovascular			Medical Care
Leidy et al. [34]	1999	General		Yes	Value in Health
Osoba [35]	1999	Oncology			European Journal of Cancer
de Haes et al. [36]	2000	Oncology			European Journal Cancer
Revicki et al. [37]	2000	General		Yes	Quality of life Research
Bottomley [38]	2001	Oncology			Applied Clinical Trials
Hakamies-Blomqvist et al. [39]	2001	Oncology			Journal of Advanced Nursing
Santanello et al. [40]	2002	General		Yes	Value in Health
Chassany et al. [41]	2002	General	Yes	Yes	Drug Information Journal
EORTC QLG [42]	2002	Oncology			Guidance document
Movsas [43]	2003	Oncology			Seminars in Radiation Oncology
Calvert & Freemantle [44]	2004	General	Yes		Journal of Clinical Pharmacy and Therapeutics
Wiklund [45]	2004	General	Yes	Yes	Fundamental & Clinical Pharmacology
Buchanan et al. [46]	2005	Oncology			Journal of Clinical Oncology
Fayers & Hays [47]	2005	General			Book (Chapter 3.2)
Lipscomb [48]	2005	Oncology			Book (Fairclough Chapter)
Avery & Blazeby [49]	2006	Oncology			World Journal of Surgery
TRoG [50]	2007	Oncology			Policy document
Ganz & Gotay [51]	2007	Oncology			Journal of Clinical Oncology
Lipscomb et al. [52]	2007	Oncology			Journal of Clinical Oncology
Land et al. [53]	2007	Oncology			Journal of Clinical Oncology
Patrick et al. [54]	2007	General		Yes	Value in Health
Sloan et al. [55]	2007	General		Yes	Value in Health
Revicki et al. [56]	2007	General		Yes	Value in Health
Fayers & Machin [57]	2007	General	Yes		Book
FDA [1]	2009	General		Yes	Guidance document
Fairclough [58]	2010	General	Yes		Book
Hao [59]	2010	Oncology		Yes	Expert Reviews
NCIC CTG [60]	2010	Oncology	Yes		Guidance document
Basch et al. [61]	2011	Oncology			Guidance document
King [66]	2011	Oncology	Yes		Web-based guidance document
Efficace & Taphoorn [62]	2012	Oncology			Journal of Neurooncology
Jensen et al. [63]	2012	Oncology			Clinical Investigation
Novik et al. [64]	2012	Haematology			Guidance document
Macefield et al. [65]	2013	Oncology			British Journal of Surgery
Kyte et al. [67]	2013	General			JAMA

*protocol guidance makes reference to the FDA or EMA.

### PRO Protocol Guidance

The included guidance documents contained 162 unique recommendations regarding PRO study design/conduct information that should be included in trial protocols. There was disagreement between data extractors on the inclusion of 10 items (6.2%), 6 of these items were included following discussion by the team, whilst 4 were felt to be duplicates of existing items. Of the 162 included items, 134 recommendations were explicit (e.g. ‘All analyses should be clearly defined a priori in the research protocol’ [41]), and 28 were implicit (e.g. ‘…investigators need to provide a rationale for the selection of a particular HRQL instrument’ [46]). Protocol recommendations are summarised in Table 2 and presented in full in Appendix S2; in both places, they are grouped under SPIRIT guidance headings, with additional subheadings used to organise content. In addition, 10 PRO recommendations were related to other supporting trial documentation (Appendix S3).

**Table 2:** Recommendations appearing in guidance documents.

Recommendation	Number (%) of Guidance Documents
**ADMINISTRATIVE INFORMATION**	
**Roles & Responsibilities of PRO Personnel**	
Name the QOL sub-study coordinator - include contact details & institution	**2 (3.70)**
Research nurses should be involved in protocol development	**1 (1.85)**
Name the QOL sub-study manager/assistant/officer - include contact details & institution	**1 (1.85)**
*The involvement of personnel other than those listed in the study should be specified (e.g. Psychologists, social workers etc)	**1 (1.85)**
**INTRODUCTION**	
**Background, Rationale & Objectives**	
**Background PRO-specific information**	
Describe what is currently known about QOL in this area and explain the gaps in literature	**5 (9.26)**
*Describe the population of interest	**3 (5.56)**
**PRO-specific rationale**	
Provide a rationale for measuring QOL- e.g. superior intervention/negative impact of intervention/equivalence	**26 (48.15)**
Provide a clinical justification for QOL outcome measurement	**12 (22.22)**
Emphasise importance of QOL assessment to the study	**6 (11.11)**
Describe why PROs have been included appropriate to the study population, intervention, context objectives and setting	**5 (9.26)**
Justify the relevance of assessing HRQL for disease and population under investigation	**5 (9.26)**
**Pro-specific hypotheses/objectives**	
State the QOL hypothesis (and corresponding null hypothesis) and to which outcome the hypothesis relates	**23 (42.59)**
Identify QOL as an objective/state research objective of HRQL component in relation to dimensions, population and timeframe	**22 (40.74)**
*Protocols addressing comparative effectiveness research in oncology should include Core Outcome Set symptoms	**2 (3.70)**
*State whether the study is exploratory or confirmatory.	**1 (1.85)**
**METHODS: PARTICIPANTS, INTERVENTIONS AND OUTCOMES**	
**PRO study setting**	
*Description and rationale of sampling method (representativeness of population and/or centres, as appropriate)	**3 (5.56)**
**PRO eligibility criteria**	
Specify if QOL completion is a pre-randomisation eligibility condition	**18 (33.33)**
*Describe patient eligibility criteria	**8 (14.81)**
State the inclusion/exclusion criteria for QOL endpoint(s) and analyses (e.g., language/reading requirements)	**7 (12.96)**
**PRO endpoint specification**	
Define the role of the PRO endpoint (primary, important secondary, exploratory)	**14 (25.93)**
Specify the timeframe of interest/primary time-point for analysis and the rationale for this	**14 (25.93)**
Identify QOL as an endpoint	**5 (9.26)**
Include a conceptual model to define exactly what is being measured,which domains are covered and what is the intended HRQL claim	**5 (9.26)**
Describe the constructs used to evaluate the intervention e.g. overall QOL, specific domain, specific symptom	**4 (7.41)**
**Timing of PRO assessments**	
Specify if baseline assessment is pre-randomisation	**19 (35.19)**
Specify acceptable time windows for each assessment	**16 (29.63)**
Specify standardised timing of questionnaire delivery (e.g. before/whilst/after seeing clinician)	**16 (29.63)**
Include QOL assessment timings in main protocol schedule of assessments	**7 (12.96)**
Outline standardised order for administration of PRO and clinical assessments	**2 (3.70)**
Specify which measures will be used at each assessment	**1 (1.85)**
For open label trials - PRO instruments administered in a clinic visit should beadministered before other clinical assessments or procedures	**1 (1.85)**
**Justification for timing of PRO assessments**	
Specify the timing of QOL assessments - link to hypotheses	**37 (68.52)**
Timing should link to research questions, length of recall,disease/Tx natural history, planned analysis/must be fair for both arms	**9 (16.67)**
*Long-term assessments in different treatment groups should be at similar times in relationto the date of randomisation to avoid bias	**3 (5.56)**
**Other timing information**	
Minimum assessment at baseline, end of study or at withdrawal	**5 (9.26)**
Conduct clinical and QOL assessment simultaneously	**3 (5.56)**
**PRO sample size**	
State the sample size and power requirements in relation to the rationale/objectives/hypothesis	**22 (40.74)**
If the sample size required for HRQL assessment is substantially less than for primary endpoint an unbiased strategy for selectionof a subset of patients in whom HRQL will be assessed is possible provided that this strategy is clearlydefined and justified in the protocol	**4 (7.41)**
**PROs in relation to blinding**	
In a blinded study detail the use of PRO administration techniques to minimise the possibility of unblinding	**1 (1.85)**
Research protocol must specify that interviewers be blind to intervention	**1 (1.85)**
**METHODS: DATA COLLECTION, MANAGEMENT AND ANALYSIS**	
**METHODS: DATA COLLECTION**	
**PRO instrument description**	
Describe the questionnaire(s) (including, number of items/domains, instrument scaling/scoring, reliability,content and construct validity, responsiveness, sensitivity, respondent burden,cultural adaptation/validity, recall period)+/− validation plan if appropriate	**19 (35.19)**
Number of items and domains	**1 (1.85)**
Detail availability of instrument in different languages and their use on the study	**1 (1.85)**
If appropriate describe administering different QOL forms to subgroups of patients	**1 (1.85)**
**PRO instrument justification**	
Specify which QOL questionnaires will be used - link to clinical justifications and hypotheses via specific domains/items	**32 (59.26)**
Specify the HRQL domains the study intervention is expected to effect	**17 (31.48)**
Reference the validity, reliability and responsiveness of the instrument (may be more succinct with refs if PROM widely used)	**14 (25.93)**
Outline plans for validation of measurement properties, if appropriate	**7 (12.96)**
Describe PROM recall period - link to treatment effects	**5 (9.26)**
Describe questionnaire completion time	**4 (7.41)**
Selection of questionnaire should be discussed and justified	**3 (5.56)**
Discuss respondent burden	**2 (3.70)**
Provide evidence that questionnaire is acceptable to patients	**2 (3.70)**
International trials should include cultural validity of questionnaire, documentation of any procedures/eventsthat differ across countries, analysis of cross culture equivalence	**2 (3.70)**
Specify why the particular questionnaire was chosen in preference to others	**1 (1.85)**
Provide evidence of measurement equivalence across modes (when mixing modes of PRO data collection)	**1 (1.85)**
Justify use of questionnaires that take longer than 10 mins to complete (or 20 mins at baseline)	**1 (1.85)**
**PRO data collection plan**	
Specify how QOL will be assessed - pencil and paper, online, etc	**22 (40.74)**
Include a pre-specified data collection plan	**17 (31.48)**
Specify if help and or proxy assessments are permitted (and what level of assistance allowed)	**14 (25.93)**
Specify where QOL will be assessed - clinic, home, etc	**12 (22.22)**
Ensure privacy and confidentiality of planned data collection	**3 (5.56)**
Specify location (e.g. quiet area [an example but not formal recommendation])	**2 (3.70)**
Specify how patients will be managed if translations unavailable	**1 (1.85)**
Specify whether the QOL instrument will be used in other languages – if so, which	**1 (1.85)**
Substantiate use of proxies (conditions under which proxy permissible)	**1 (1.85)**
State who will administer the measure (e.g., a physician, nurse etc)	**1 (1.85)**
**PRO data collection guidelines/training infromation**	
Provide guidelines and/or training plan for PRO data collection	**36 (66.67)**
Specify that a named person at each centre (and/or centrally?) be nominated to take responsibility for admin,collection and checking of QoL forms - specify whether this is or is not the treating clinician	**27 (50.00)**
Provide instructions on how the patient should complete the form (e.g. without conferring with friends/relatives,all questions should be answered even if the patient feels them to be irrelevant)	**21 (38.89)**
Specify procedures for checking questionnaires/prevention of avoidable missing data, who will check form, and how will they deal with missing questionnaire(s) or items	**21 (38.89)**
Emphasise importance of good compliance/describe procedures to maximise compliance/minimise missing data	**20 (37.04)**
Encourage staff to emphasise importance/rationale of QOL assessment to patients	**13 (24.07)**
Provide reminders to staff to ensure baseline (and follow-up) questionnaires are completed	**11 (20.37)**
Pre-specified procedures in protocol to avoid/minimise missing data	**8 (14.81)**
Establish process for PRO assessment at withdrawal for patients that withdraw early from a study	**7 (12.96)**
Establish process of how to follow patients who go”off-study”/"off treatment”	**7 (12.96)**
Provide interviewer training plan/format guidelines for PROs administered by interviewer (plus guidance on recording of interviews)	**6 (11.11)**
Specify need to ensure backup data collection staff to cover leave/absence	**6 (11.11)**
A plan should be included in protocol for systematically training and contacting local site personnel to ensure that they understand the content and importance of collecting PRO data. Ideally coordinated by a lead data manager who monitors patient adherence in real time and communicates with sites if patient non-adherent	**5 (9.26)**
Instruct site staff to routinely record (on clinical follow-up form) the reasons for any missing data	**4 (7.41)**
Explain relevance and emphasise importance of QOL questions that might give rise to problems (e.g. sexual function questions)	**4 (7.41)**
Detail/outline site-level incentives for good QOL submission rates/data quality and penalties for missing data (as appropriate)	**4 (7.41)**
Encourage a sympathetic approach to patients who may be feeling particularly ill/show appreciation upon completion	**4 (7.41)**
Instruct site staff to give patients a full explanation about QoL assessment procedures	**3 (5.56)**
Instruct site staff to routinely record the source of PRO data in studies that allow proxies	**3 (5.56)**
Establish back up plans for gathering treatment-related reasons for patients failing to report at scheduled times	**3 (5.56)**
Instruct site staff to routinely record (on clinical follow-up form) whether QOL assessment completed	**2 (3.70)**
Instruct site staff to routinely record (on clinical follow-up form) if the patient needed help to complete the questionnaire	**2 (3.70)**
Provide online training and state in the protocol how this will be accessed	**2 (3.70)**
Describe process for certification (and re-certification) for staff conducting PRO assessment	**2 (3.70)**
Specify procedures for continuous QOL instruction/training of staff (needed due to staff changes)	**2 (3.70)**
Include guidance on discussing importance of PROs with patient	**2 (3.70)**
Instruct staff regarding the importance of including QOL assessment alongside regular data collection	**2 (3.70)**
Ensure patients understand the schedule for and importance of follow up visits	**2 (3.70)**
Encourage the patient to request their QOL forms upon arrival at the clinic	**2 (3.70)**
Specify procedures for minimising inconsistencies in trial conduct	**1 (1.85)**
Provide training plan and instructions to patients for self-administered PROs	**1 (1.85)**
Instruct data collection staff to record the specific mode of PRO administration (in studies with mixed modes of PRO data collection)	**1 (1.85)**
Provide instructions for clinical investigators regarding patient supervision	**1 (1.85)**
Include details on data collection and management methods to minimise missing data	**1 (1.85)**
Strategies for minimising the exclusion of subjects from the trial	**1 (1.85)**
**Plans to avoid/minimise missing data**	
**METHODS: DATA MANAGEMENT**	
**PRO-specific Quality Assurance**	
Specify procedures for a central PRO data monitoring system (aimed at identifying and rectifying potential data collection problems)	**13 (24.07)**
Specify plan to monitor compliance	**7 (12.96)**
Include data collection, data storage and data handling/transmission procedures	**5 (9.26)**
Specify how an electronic PRO source will be maintained and how investigator will meet regulatory requirements and ensure data integrity and security	**2 (3.70)**
Specify procedure for monitoring adherence to timing windows'	**2 (3.70)**
Include guidance for data entry on coding responses, missing responses or ambiguous responses	**1 (1.85)**
Ensure plans for administration consistent with user manual	**1 (1.85)**
**METHODS: DATA ANALYSIS**	
**PRO Statistical Analysis**	
All analyses should be clearly defined a priori in the protocol	**29 (53.70)**
Specify ITT or per-protocol analysis.	**6 (11.11)**
Pre-specify scoring	**6 (11.11)**
State the anticipated response rate/effect size	**4 (7.41)**
Specify conditions for positive outcome	**3 (5.56)**
Ensure plans for scoring are consistent with those used in development	**3 (5.56)**
Describe methods for scoring endpoints. Where possible, reference scoring manuals for summated scales from questionnaires (domain-specific and/or total),and methodological papers for composite endpoints (e.g. QTWiST)	**2 (3.70)**
Explain the assumptions of analyses	**1 (1.85)**
Include an a priori estimation of expected change in PRO score	**1 (1.85)**
Include a priori identified summary statistics (as appropriate)	**1 (1.85)**
Specify minimum amount of QOL data and acceptable degree of timing deviation before compromise of study question	**1 (1.85)**
Describe approach to controlling for QOL related comorbidity	**1 (1.85)**
Include appropriate procedures for minimising assessment bias	**1 (1.85)**
**Plans to address multiplicity of PRO data**	
Plan for multiplicity/controlling type 1 error - summary measures/adjustments	**19 (35.19)**
Pre-specification of sequence of testing (regulatory trials)/exploratory analyses to control for multiplicity or prespecify domains for a labelling claim	**7 (12.96)**
**PRO clinical significance**	
State and justify minimal [clinical] important difference/change	**11 (20.37)**
Specify the criteria for statistical and clinical significance	**4 (7.41)**
Define clinical response/method of analysis for response/cumulative distribution function	**4 (7.41)**
Identify and state score change meaningful to patient	**2 (3.70)**
Describe QOL responder definitions (size and duration of benefit) where relevant	**1 (1.85)**
Investigators should indicate how the results will be used.	**1 (1.85)**
**Statisitical methods to deal with missing PRO data**	
Describe methods for handling missing data	**22 (40.74)**
Include proposed sensitivity analyses for imputation methods.	**6 (11.11)**
Include approach to imputation	**4 (7.41)**
State how missing data will be described	**3 (5.56)**
**MONITORING**	
**PRO data monitoring**	
Role of DMC and QA in relation to PROs should be defined.	**12 (22.22)**
**PRO alerts**	
Specify mechanism for alerting clinical staff about symptoms reported by patients that exceed a pre-defined level of severity	**2 (3.70)**
Provide guidance for staff on where they should refer patients for appropriate help, should completion of the QoL questionnaire prompt them to seek more information or support	**1 (1.85)**
Include an a priori plan for consistent/standardised management of PRO alerts that is clearly communicated to all appropriate trial staff	**1 (1.85)**
**ETHICS AND DISSEMINATION**	
**PRO-specific consent information**	
Explain the QOL assessment procedure within the PIS/consent (including: reasons for evaluating QoL, what it will involve, risks and benefits, frequency and timing/timeframe, the need to answer all questions, the importance of completing questions without being influenced by the opinions of others) and, if appropriate, identify if consent to QOL assessment is required for entry into the trial	**14 (25.93)**
*Describe informed consent procedure.	**1 (1.85)**
Inform patients who they may contact for help in completing the questionnaire	**1 (1.85)**
**Pro-specific confidentiality procedures**	
Include guidance on discussing PRO confidentiality with patients (e.g. patients told how their questionnaires will be used)	**9 (16.67)**
Specify whether QOL forms will be used to influence therapy or patient management (i.e. will clinician have knowledge)	**4 (7.41)**
**PRO dissemination policy**	
Include detailed plans for regular feedback to participants via letter/newsletter on QOL aspect of study	**2 (3.70)**
Include QOL publication policy	**1 (1.85)**
**APPENDICES**	
**PRO components of informed consent materials**	
A sample PIS and consent form (in which QOL assessment requirements are mentioned) should be included in an appendix of the protocol	**8 (14.81)**
PIS/Consent form should inform patients what will happen to their completed questionnaires	**5 (9.26)**
Patients should be provided with QOL information leaflet to take home	**2 (3.70)**
**PRO assessment checklist and/or flowsheet**	
Include statistical analysis plan in protocol appendix	**3 (5.56)**
Include a QOL Assessment checklist in a protocol appendix	**2 (3.70)**
Details about the characteristics of the PRO should be included in an appendix	**1 (1.85)**
Formal statement on QOL data collection policy should be included in the appendix	**1 (1.85)**
Checklist should be provided in the protocol	**1 (1.85)**
Include a QOL patient evaluation flow sheet in the appendix	**1 (1.85)**
**PRO questionnaire**	
Provide exact version/format of PROM in the protocol within the CRF (or in a separate appendix as appropriate)	**10 (18.52)**
Present evidence of permission to use QOL questionnaire (where applicable)	**2 (3.70)**
Include translations of core questionnaire in appendix	**1 (1.85)**
**PROM completion instructions**	
Ensure clear written patient instructions accompany QOL/PRO questionnaire	**9 (16.67)**
**General Referencing**	
References should be provided to support key statements	**2 (3.70)**
**GENERAL APPROACH TO PROTOCOL**	
PRO endpoints should be fully integrated in the trial protocol/data collection	**33.33%**
PROs should be addressed in a separate chapter in the protocol	**7.41%**
Relevant sections pertaining to QOL should be identified in the protocol table of contents	**1.85%**

*These recommendations were made specifically in relation to the PRO component of the study.

### Administrative information

There were n = 4 recommendations regarding trial administration, which centred around identifying the roles and responsibilities of PRO personnel and ranged from advocating involvement of the research nurse in PRO protocol development, to providing the contact details of the Quality of Life (QOL) sub-study coordinator where appropriate.

### Introduction: Background, rationale, and objectives/hypotheses

Eleven unique recommendations related to the inclusion of PROs in the introductory sections of the protocol. These focused on aspects surrounding: PRO specific background information (n = 2), for instance, the need to describe the PRO population of interest; specification of the PRO rationale (n = 5), for example, justifying the relevance of PRO assessment in the disease and population under investigation; or outlining the PRO hypothesis and objectives (n = 4).

### Methods: Participants, interventions and outcomes

There were n = 25 unique recommendations within this section, focused on a number of areas, including: the PRO study setting (n = 1), the PRO-specific eligibility criteria (n = 3), the need to specify the PRO as an endpoint (n = 5), the PRO-specific sample size (n = 2) and blinding considerations (n = 2). Twelve different recommendations related to timing of the PRO assessment, ranging from: including PRO assessment timings in the main protocol assessment schedule and specifying time windows, to justifying timings according to the study research questions, length of recall of the questionnaire, the natural history of the disease under study, and any planned analysis.

### Methods: assignment of interventions

There were no PRO-specific recommendations identified under this heading.

### Methods: Data Collection, management and analysis

Ninety-four recommendations related to PRO-specific protocol guidance for data collection, management and analysis. These focused on data collection aspects including: identification/description of the PRO instrument (n = 4), for instance, the need to outline the questionnaire domains and number of items; justifying the choice of instrument (n = 13), for example, the importance of referencing the validity, reliability and responsiveness of the tool; detailing the data collection plan (n = 10), for example, stating who should administer the questionnaire; and describing the data collection/training guidelines (n = 16) (e.g. outlining the certification process for staff involved in PRO assessment) and plans to minimise missing data (n = 19) for example specifying who would check questionnaires for missing data. There were n = 7 recommendations concerning PRO specific quality assurance, ranging from the inclusion of guidance for data entry coding decisions regarding missing or ambiguous responses; to specifying procedures for a central PRO data monitoring system aimed at identifying and rectifying potential data collection problems. Finally, n = 13 recommendations focused on PRO analysis, specifically: the PRO-specific components of the statistical analysis plan (n = 13), for instance, the need to include an a priori estimation of expected change in PRO score; plans to address multiple hypothesis testing (n = 2), such as pre-specification of sequence of testing; defining clinical significance (n = 6), for example, describing and justifying the minimal clinically important difference/change); and specifying methods to deal with missing PRO data (n = 4), for instance, defining proposed sensitivity analyses for imputation methods.

### Methods: Monitoring

There were four recommendations regarding PRO specific trial monitoring, ranging from the need to define the role of the Data Monitoring Committee in relation to PROs, to the inclusion of a plan to manage PRO Alerts.

### Ethics and Dissemination

There were n = 3 recommendations focused on PRO-specific consent information, for example, the need to include information for patients regarding who should be contacted for help with completing the PRO questionnaire. Two recommendations addressed PRO specific confidentiality issues, such as the need to specify whether QOL data will be used to influence patient management. Two recommendations focused on the need to include PRO-specific dissemination plans, through both peer-reviewed scientific publication and direct participant contact.

### Appendices

Fourteen recommendations focused on the inclusion of relevant PRO documents as protocol appendices, including: a copy of the PRO questionnaire(s), sample patient information and consent materials containing PRO information and a PRO-specific administration flow chart/checklist.

### Other Trial Documentation

Ten recommendations focused on PRO information that should be included in protocol-related trial documents such as Standard Operating Procedures (SOPs), Case Report Forms (CRFs) or training manuals (Appendix S3).

### Time trends and Common Recommendations

**Figure 2:**
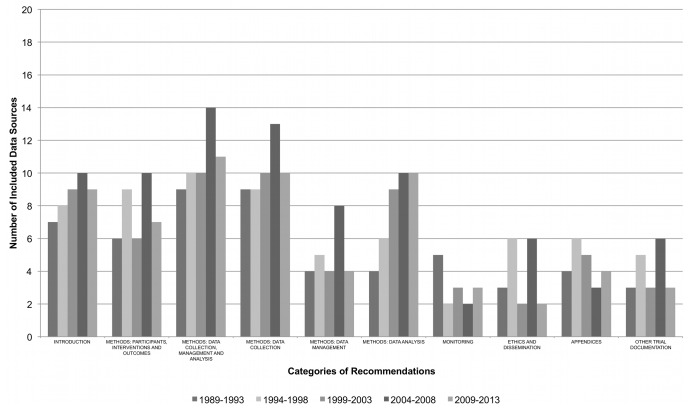
PRO protocol guidance trends over time.

The availability of PRO-specific guidance over time is shown in Figure 2. The data suggest that there has been consistent publication of PRO protocol guidance, across all areas, over the last 25 years (Table 3). In addition, over 75% of recommendations extracted for this study have been available for at least 10 years.

**Table 3:** PRO protocol guidance over time.

Time Period	N (%) of new PRO Protocol Recommendations
1989–1993	70 (43.21)
1994–1999	30 (18.52)
1999–2003	23 (14.20)
2004–2008	9 (5.56)
2009–2013	30 (18.52)

Only 3% of recommendations appeared in more than half of the documents included in the study, highlighting a lack of consistency in the PRO guidance literature reviewed (Table 2). These included (in order of frequency): the need to specify the timing of QOL assessment, the provision of PRO data collection guidelines and/or a training plan, specification (and justification) for the chosen PRO questionnaire, routine inclusion of *a priori* defined PRO analyses plans and specifying a named person within the trial with responsibility for overseeing QOL assessment.

## Discussion

### Summary of Findings

Our review is the first to summarise the current PRO-specific guidance for clinical trial protocol developers. In total we identified 54 guidance documents[1], [15]–[67], which provided 162 recommendations regarding PRO-specific information that should be included in protocols containing a PRO endpoint.

Unfortunately, although PRO protocol guidance has been in existence for over 25 years, our findings suggest it remains difficult to implement in practice. First, with the exception of 8 publications[16], [22], [39]–[41], [59], [62], [63] sourced via electronic database searches, the guidance literature was particularly difficult to access. The remaining 46 documents, which provided more than half (56.7%) of all PRO protocol recommendations, were obtained via citation tracking, hand-searching reference lists of included articles, grey literature review and expert contact. It is unlikely that protocol developers would have the time or resources to carry out such a comprehensive search. As such, developers may be reliant on a small proportion of guidance documents available via easily accessible scientific databases. This is problematic, as these publications provide relatively little coverage of the current PRO protocol recommendations in circulation. As our findings show, recommendations are spread over a wide variety of sources, thus, over reliance on a small number of guidance documents may mean important PRO design considerations are overlooked. For example, even the two publications that provided most recommendations, Chassany [41] and Fairclough [58] (42 recommendations each, 24 shared), provided just 37.04% of the total in circulation.

Second, developers wishing to use the guidance summarised in this review face the challenge of trying to incorporate a large number of recommendations into what is usually a rather limited amount of space within the protocol. For instance, we identified 94 unique recommendations concerning data collection, management and analysis, of which, 19 alone addressed minimising missing data. Tackling missing PRO data is clearly an important design consideration since it helps reduce bias and preserves statistical power [68], however, it may be unrealistic to expect protocol developers to incorporate all 19 recommendations within a study protocol.

It is important, therefore, for the scientific community to reach consensus on the essential PRO protocol content required to preserve trial integrity; and to provide guidance that is useful in practice. We advocate the development of consolidated, easily accessible and internationally endorsed consensus guidelines addressing this objective. Our review provides a useful starting point as it presents a comprehensive list of the PRO protocol guidance currently available, however, it remains unclear at this stage exactly which of the recommendations identified in this study should be incorporated into consolidated guidelines. A number of recommendations are supported by multiple sources and appear to be underpinned by a clear theoretical justification (for example, the need to provide a rationale for PRO measurement (recommended in 48.15% of guidance documents)), and may be promising candidates for inclusion. There were, however, a number of other recommendations that were less frequently cited, but still may have important implications for trial conduct, reporting and the quality of PRO results. For example, referencing the PRO instrument validity and reliability in the protocol (recommended in 25.93% of guidance documents) will help ensure that the psychometric properties of the PRO have been duly considered during the trial design and will help facilitate later reporting in accordance with the CONSORT-PRO extension. [69] In addition, only four publications provided guidance describing plans for the identification and management of PRO Alerts, that is: ‘concerning levels of psychological distress or physical symptoms that may require an immediate response’ [67]. However, evidence suggests that without clear, pre-specified, plans for the management of PRO Alerts, either in the trial protocol or supporting documentation, variation may occur in their management within/across trial sites risking co-intervention bias and suboptimal patient care. [67].

Consolidated PRO guidance for protocol developers is clearly necessary, however, our findings also show that guidelines should be developed using robust consensus methodology to ensure that the merits of all individual recommendations are carefully considered prior to selection/rejection. The definitive guidelines should aim to improve the quality of PRO trial design and reporting, resulting in more robust PRO trial data that will exert a greater influence on clinical practice and will provide an improved information base for future patients. Researchers should be supported in implementing the guidance through training and online resources. Furthermore, endorsement by funding bodies and Institutional Review Boards/Ethical Committees, who review the content of protocols, and journal editors, who are responsible for their publication, is important to ensure widespread adoption.

### Strengths and Limitations

Our review has for the first time collated and summarised the existing PRO guidance available for protocol developers using systematic methods and multiple reviewers. A limitation of our approach is that the PRO item categorisation and indexing employed during our analysis is influenced by reviewer interpretation. Also, publications included in the study had to provide guidance on PRO-protocol content; however, such guidance was not always the main focus or aim of some of the included articles. Again, the interpretation of the reviewer may subtly alter the original meaning of the text drawn from such material. The use of independent dual data extraction by 2 investigators (with a third to mediate) sought to reduce these effects, however, they remain a legitimate concern. Furthermore no formal quality appraisal was undertaken given the diverse nature of the guidance documents. Relevant PRO guidance literature was difficult to source and appeared to be particularly poorly indexed. Whilst we employed a number of resources to comprehensively search the literature (including electronic databases, citation tracking and hand searching, internet search engines and expert contact) further PRO guidance probably exists that was not included in our study, such as disease specific documents citing EMA and FDA guidance included in our review. Consideration of alternative search strategies, such as ‘citation pearl methodology’ [70], may have further increased the comprehensiveness of our review. The International Society for Quality of Life Research Best Practice for PROs in Trials Taskforce (Co-chaired by authors, MC, MK and MB) is leading work in this area and would value the identification of further grey literature across a range of clinical settings on this topic by interested readers to inform future guideline development. A further limitation is that most of the international advisory group work in an oncology setting therefore are likely to have greater knowledge of grey literature in this field. However, oncologists have routinely used PROs for many years and as such we may anticipate more literature in this clinical area.

### Conclusion

PRO-specific protocol guidance is difficult to access, lacks consistency and is unwieldy; with over 160 recommendations spread across 54 different publications. It is therefore extremely challenging to implement in practice. There is a need to develop easily accessible consolidated, and consensus-driven, PRO protocol guidelines. Guidance should aim to ensure key PRO information is routinely included in trial protocols with a PRO endpoint, in order to facilitate the rigorous collection and reporting of PRO data, thus maximising its capacity to effectively inform patient care.

## Supporting Information

Checklist S1 **PRISMA Checklist.**
(DOC)

Appendix S1 **Full search strategy.**
(DOCX)

Appendix S2 **Protocol recommendations by source publication.**
(XLSX)

Appendix S3 **PRO recommendations relating to other supporting trial documentation.**
(XLSX)
