# Supplementary material for: Patient-Reported Outcome (PRO) Assessment in Clinical Trials: A Systematic Review of Guidance for Trial Protocol Writers
Source: PLoS One. 2014 Oct 15;9(10):e110216. doi: 10.1371/journal.pone.0110216 (PMC4198295; doi:10.1371/journal.pone.0110216)
Supplement: Appendix S1 — Full search strategy. (DOCX) [file pone.0110216.s002.docx]

**Appendix I - Search Strategy**

*Eligibility criteria:*

Papers must provide (1) Guidelines/Checklist, on (2) PRO-related, (3) trial protocol content.

*Databases:*

MEDLINE (OVID), EMBASE, CINHAL, COCHRANE LIBRARY

**Search Strategy (MEDLINE)**

| **MEDLINE** |
| --- |
| **PRO terms** |
| 1) Patient reported outcome*.tw. |
| 2) Self-reported outcome*.tw. |
| 3) exp "Quality of Life"/ |
| 4) Patient Satisfaction/ |
| 5) adherence.mp. |
| 6) Fatigue/ |
| 7) exp *Health Status/ |
| 8) "Activities of Daily Living"/ |
| 9) life qualit$.tw. |
| 10) exp self concept/ |
| 11) health level.tw. |
| 12) level of health.tw. |
| 13) wellness.tw. |
| 14) well being.tw. |
| 15) (activities of daily life or daily living activities).tw. |
| 16) functional ability.tw. |
| 17) good health.tw. |
| 18) healthiness.tw. |
| 19) social adjustment/ |
| 20) physical limitations.tw. |
| 21) psychiatric status.tw. |
| 22) pain measurement/ |
| 23) functional assessment.tw. |
| 24) QoL.tw. |
| 25) hrql.tw. |
| 26) hrqol.tw. |
| 27) exp *"Outcome Assessment (Health Care)"/ |
| 28) health status.tw. |
| 29) lifestyle.tw. |
| 30) questionnaire*.tw. |
| 31) symptom assessment.tw. |
| 32) needs assessment.tw. |
| 33) quality of life.tw. |
| 34) exp *Questionnaires/ |
| 35) (patient$ adj2 reported).tw. |
| 36) self report$.tw. |
| 37) patient$ experience$.tw. |
| 38) PROM$1.tw. |
| 39) *Pain/ |
| 40) Pain, Postoperative/ |
| 41) *"Severity of Illness Index"/ |
| 42) wellbeing.tw. |
| Invalid term so removed as covered elsewhere |
| 43) Health Utility.tw. |
| 44) Health Status/ |
| [45) psychosocial.tw.](http://psychosocial.tw/) |
| [46) psycho-social.tw.](http://psycho-social.tw/) |
| 47) exp Patient Satisfaction/ |
| 48) (outcome$ adj5 expectation$).tw. |
| 49) (outcome$ adj5 satisfaction).tw. |
| 50) (outcome$ adj5 (satisfaction or satisfied)).tw. |
| 51) "Interviews as Topic"/ |
| 52) (symptom$ adj5 (improv$ or change$ or deteriorat$)).tw. |
| 53) (patient$ adj5 priorit$).tw. |
| 54) (scale or scales).tw. |
| [55) expectations.tw.](http://expectations.tw/) |
| [56) satisfaction.tw.](http://satisfaction.tw/) |
| 57) "Recovery of Function"/ |
| 58) Or/1-57 |
|  |
| **Guideline/checklist terms** |
| 59) exp Guideline/ |
| 60) exp Practice Guideline/ |
| 61) (Guidance or Recommendation* or Standard*).mp |
| 62) exp Checklist/ |
| 63) exp Medical Ethics/ |
| 64) exp Research Ethics/ |
| 65) exp informed consent/ |
| 66) Professional Obligation.tw. |
| 67) Duty of Care.tw. |
| 68) Or/59-67 |
|  |
| **Trial Protocol terms** |
| 69) (Trial design or study design).mp |
| 70) (Trial Protocol* or study protocol*).mp |
| 71) Or/69-70 |
| 72) And /58, 68,71 |

**Search Strategy (EMBASE)**

| **EMBASE** |
| --- |
| **PRO terms** |
| 1) Patient reported outcome*.tw. |
| 2) Self-reported outcome*.tw. |
| 3) exp "Quality of Life"/ |
| 4) Patient Satisfaction/ |
| 5) adherence.mp. |
| 6) Fatigue/ |
| 7) exp *Health Status/ |
| 8) "Activities of Daily Living"/ |
| 9) life qualit$.tw. |
| 10) exp self concept/ |
| 11) health level.tw. |
| 12) level of health.tw. |
| 13) wellness.tw. |
| 14) well being.tw. |
| 15) (activities of daily life or daily living activities).tw. |
| 16) functional ability.tw. |
| 17) good health.tw. |
| 18) healthiness.tw. |
| 19) social adjustment/ |
| 20) physical limitations.tw. |
| 21) psychiatric status.tw. |
| 22) pain measurement/ |
| 23) functional assessment.tw. |
| 24) QoL.tw. |
| 25) hrql.tw. |
| 26) hrqol.tw. |
| 27) exp *"Outcome Assessment (Health Care)"/ |
| 28) health status.tw. |
| 29) lifestyle.tw. |
| 30) questionnaire*.tw. |
| 31) symptom assessment.tw. |
| 32) needs assessment.tw. |
| 33) quality of life.tw. |
| 34) exp *Questionnaires/ |
| 35) (patient$ adj2 reported).tw. |
| 36) self report$.tw. |
| 37) patient$ experience$.tw. |
| 38) PROM$1.tw. |
| 39) *Pain/ |
| 40) Pain, Postoperative/ |
| 41) *"Severity of Illness Index"/ |
| 42) wellbeing.tw. |
| 43) Health Utility.tw. |
| 44) Health Status/ |
| [45) psychosocial.tw.](http://psychosocial.tw/) |
| [46) psycho-social.tw.](http://psycho-social.tw/) |
| 47) exp Patient Satisfaction/ |
| 48) (outcome$ adj5 expectation$).tw. |
| 49) (outcome$ adj5 satisfaction).tw. |
| 50) (outcome$ adj5 (satisfaction or satisfied)).tw. |
| 51) "Interviews as Topic"/ |
| 52) (symptom$ adj5 (improv$ or change$ or deteriorat$)).tw. |
| 53) (patient$ adj5 priorit$).tw. |
| 54) (scale or scales).tw. |
| [55) expectations.tw.](http://expectations.tw/) |
| [56) satisfaction.tw.](http://satisfaction.tw/) |
| 57) "Recovery of Function"/ |
| 58) Or/1-57 |
|  |
| **Guideline/checklist terms** |
| 59) exp Guidelines/ |
| 60) exp Practice Guideline/ |
| 61) (Guidance or Recommendation* or Standard*).mp |
| 62) exp Checklist/ |
| 63) exp Medical Ethics/ |
| 64) exp Research Ethics/ |
| 65) exp informed consent/ |
| 66) Professional Obligation.tw. |
| 67) Duty of Care.tw. |
| 68) Or/59-67 |
|  |
| **Trial Protocol terms** |
| 69) (Trial design or study design).mp |
| 70) (Trial Protocol* or study protocol*).mp |
| 71) Or/69-70 |
| 72) And /58, 68,71 |

**Search Strategy (CINHAL)**

| **CINAHL** |
| --- |
| **PRO terms** |
| 1) (MH "Outcome Assessment") |
| 2)TI Patient reported outcome* or AB Patient reported outcome* |
| 3)(MH "Self Report") |
| 4)TI Self-reported outcome* or AB Self-reported outcome* |
| 5)(MH "Quality of Life+") |
| 6)(MH "Patient Satisfaction") |
| 7)TX adherence |
| 8)(MH "Fatigue") |
| 9)(MH "Health Status+") |
| 10)(MH "Health") |
| 11)(MH "Activities of Daily Living") |
| 12)TI life quality OR AB life quality |
| 13) (MH "Self Concept+") |
| 14)TI health level OR AB health level |
| 15)TI level of health OR AB level of health |
| 16)TI Wellness or AB Wellness |
| 17)TI well being OR AB well being |
| 18)TI activities of daily life OR AB activities of daily life |
| 19)TI daily living activities OR AB daily living activities |
| 20)TI functional ability OR AB functional ability |
| 21)TI good health OR AB good health |
| 22)TI healthiness OR AB healthiness |
| 23)(MH "Social Adjustment") |
| 24)TI physical limitations OR AB physical limitations |
| 25)TI psychiatric status OR AB psychiatric status |
| 26)(MH "Pain Measurement") |
| 27)TI Functional Assessment OR AB Functional Assessment |
| 28)TI QoL OR AB QoL |
| 29)TI hrql OR AB hrql |
| 30)TI hrqol OR AB hrqol |
| 31)(MH "Outcomes (Health Care)+") |
| 32)TI health status OR AB health status |
| 33)TI lifestyle OR AB lifestyle |
| 34)TI Questionnaire OR AB Questionnaire |
| 35)TI symptom assessment OR AB symptom assessment |
| 36)TI needs assessment OR AB needs assessment |
| 37)TI quality of life OR AB quality of life |
| 38)(MH "Questionnaires+") |
| 39)TI patient* N2 reported OR AB patient* N2 reported |
| 40)TI self report* OR AB self report* |
| 41)TI patient* experience* OR AB patient* experience* |
| 42)TI PROMS* OR AB PROMS* |
| 43)(MH "Pain") |
| 44)(MH "Postoperative Pain") |
| 45)(MH "Severity of Illness Indices") |
| 46)TI wellbeing OR AB wellbeing |
| 47)(MH "Health Resource Utilization") |
| 48)(MH "Health Resource Allocation") |
| 49)TI Health Utility OR AB Health Utility |
| 50)(MH "Health Status") |
| 51)TI psychosocial OR AB psychosocial |
| 52)TI psycho-social OR AB psycho-social |
| 53)TI outcome* N5 expectation* OR AB outcome* N5 expectation* |
| 54)TI outcome* N5 satisfaction OR TI outcome* N5 satisfaction |
| 55)( TI outcome* N5 (satisfaction or satisfied) ) OR ( AB outcome* N5 (satisfaction or satisfied) ) |
| 56)(MH "Interviews") |
| 57)( TI symptom* N5 (improv* or change* or deteriorat*) ) OR ( AB symptom* N5 (improv* or change* or deteriorat*) ) |
| 58)TI patient* N5 priorit* OR AB patient* N5 priorit* |
| 59)( TI (scale or scales) ) OR ( AB (scale or scales) ) |
| 60)TI expectations OR AB expectations |
| 61)TI satisfaction OR AB satisfaction |
| 62)(MH "Functional Status") |
| 63)(MH "Functional Assessment") |
| 64) Or/1-63 |
|  |
| **Guideline/checklist terms** |
| 65)TX Guideline* |
| 66)TI Guideline* or AB Guideline* |
| 67)(MH "Guideline Adherence") |
| 68)(MH "Practice Guidelines") |
| 69)TX Guidance |
| 70)TX Recommendation* |
| 71)TX Standard* |
| 72)(MH "Checklists") |
| 73)(MH "Ethics, Medical") |
| 74)(MH "Research Ethics+") |
| 75)(MH "Consent+") |
| 76)(MH "Consent (Research)") |
| 77)TI Professional Obligation OR AB Professional Obligation |
| 78)TI Duty of Care OR AB Duty of Care |
| 79) Or/65-78 |
|  |
| **Trial Protocol terms** |
| 80)(MH "Study Design") |
| 81)TX Trial design or study design |
| 82)(MH "Research Protocols") |
| 83)TX Trial protocol or study protocol |
| 84) Or/80-83 |
| 85) And/64,79,84 |

**Search Strategy (COCHRANE LIBRARY)**

| **Cochrane** |
| --- |
| **PRO terms** |
| 1)Patient reported outcome*:ti or Patient reported outcome*:ab (Word variations have been searched) |
| 2)Self-reported outcome*:ti or Self-reported outcome*:ab (Word variations have been searched) |
| 3)MeSH descriptor: [Quality of Life] explode all trees |
| 4)MeSH descriptor: [Patient Satisfaction] explode all trees |
| adherence (Word variations have been searched) |
| 5)MeSH descriptor: [Fatigue] explode all trees |
| 6)MeSH descriptor: [Health Status] explode all trees |
| 7)MeSH descriptor: [Activities of Daily Living] explode all trees |
| 8)life quality:ti or life quality:ab (Word variations have been searched) |
| 9)MeSH descriptor: [Self Concept] explode all trees |
| 10)health level:ti or health level:ab (Word variations have been searched) |
| 11)level of health:ti or level of health:ab (Word variations have been searched) |
| 12)wellness:ti or wellness:ab (Word variations have been searched) |
| 13)well being:ti or well being:ab (Word variations have been searched) |
| 14)(activities of daily life or daily living activities):ti or (activities of daily life or daily living activities):ab (Word variations have been searched) |
| 15)functional ability:ti or functional ability:ab (Word variations have been searched) |
| 16)good health:ti or good health:ab (Word variations have been searched) |
| 17)healthiness:ti or healthiness:ab (Word variations have been searched) |
| 18)MeSH descriptor: [Social Adjustment] explode all trees |
| 19)physical limitations:ti or physical limitations:ab (Word variations have been searched) |
| 20)psychiatric status:ti or psychiatric status:ab (Word variations have been searched) |
| 21)MeSH descriptor: [Pain Measurement] explode all trees |
| 22)functional assessment:ti or functional assessment:ab (Word variations have been searched) |
| 23)QoL:ti or QoL:ab (Word variations have been searched) |
| 24)hrql:ti or hrql:ab (Word variations have been searched) |
| 25)hrqol:ti or hrqol:ab (Word variations have been searched) |
| 26)MeSH descriptor: [Outcome Assessment (Health Care)] explode all trees |
| 27)health status:ti or health status:ab (Word variations have been searched) |
| 28)lifestyle:ti or lifestyle:ab (Word variations have been searched) |
| 29)questionnaire*:ti or questionnaire*:ab (Word variations have been searched) |
| 30)symptom assessment:ti or symptom assessment:ab (Word variations have been searched) |
| 31)needs assessment:ti or needs assessment:ab (Word variations have been searched) |
| 32)quality of life:ti or quality of life:ab (Word variations have been searched) |
| 33)MeSH descriptor: [Questionnaires] explode all trees |
| 34)patient* next reported:ti or patient* next reported:ab (Word variations have been searched) |
| 35)self report*:ti or self report*:ab (Word variations have been searched) |
| 36)patient* experience*:ti or patient* experience*:ab (Word variations have been searched) |
| 37)PROM*:ti or PROM*:ab (Word variations have been searched) |
| 38)MeSH descriptor: [Pain] explode all trees |
| 39)MeSH descriptor: [Pain, Postoperative] explode all trees |
| 40)MeSH descriptor: [Severity of Illness Index] explode all trees |
| 41)wellbeing:ti or wellbeing:ab (Word variations have been searched) |
| 42)Health Utility:ti or Health Utility:ab (Word variations have been searched) |
| 43)MeSH descriptor: [Health Status] explode all trees |
| 44) psychosocial:ti or psychosocial:ab (Word variations have been searched) |
| 45) psycho-social:ti or psycho-social:ab (Word variations have been searched) |
| 46) outcome* near/5 expectation*:ti or outcome* near/5 expectation*:ab (Word variations have been searched) |
| 47) outcome* near/5 satisfaction:ti or outcome* near/5 satisfaction:ab (Word variations have been searched) |
| 48)(outcome* near/5 (satisfaction or satisfied)):ti or (outcome* near/5 (satisfaction or satisfied)):ab (Word variations have been searched) |
| 49)MeSH descriptor: [Interviews as Topic] explode all trees |
| 50)(symptom* near/5 (improv* or change* or deteriorat*)):ti or (symptom* near/5 (improv* or change* or deteriorat*)):ab (Word variations have been searched) |
| 51) patient* near/5 priorit*:ti or patient* near/5 priorit*:ab (Word variations have been searched) |
| 52)scale or scales:ti or scale or scales:ab (Word variations have been searched) |
| 53) expectations:ti or expectations:ab (Word variations have been searched) |
| 54) satisfaction:ti or satisfaction:ab (Word variations have been searched) |
| 55) MeSH descriptor: [Recovery of Function] explode all trees |
| 56) Or/01-55 |
|  |
| **Guideline/checklist terms** |
| 57) MeSH descriptor: [Guideline] explode all trees |
| 58) MeSH descriptor: [Practice Guideline] explode all trees |
| 59) (Guidance or Recommendation* or Standard*) (Word variations have been searched) |
| 60) MeSH descriptor: [Checklist] explode all trees |
| 61) MeSH descriptor: [Ethics, Medical] explode all trees |
| 62) MeSH descriptor: [Ethics, Research] explode all trees |
| 63) MeSH descriptor: [Informed Consent] explode all trees |
| 64) Professional Obligation:ti or Professional Obligation:ab (Word variations have been searched |
| 65) Duty of Care:ti or Duty of Care:ab (Word variations have been searched) |
| 66) Or/57-65 |
|  |
| **Trial Protocol terms** |
| 67) (Trial design or study design) (Word variations have been searched) |
| 68) (Trial Protocol* or study protocol*) (Word variations have been searched) |
| 69) Or/67-68 |
| 70) And/56,66,69 |
